# Generation of Isogenic Controls for In Vitro Disease Modelling of X-Chromosomal Disorders

**DOI:** 10.1007/s12015-018-9851-8

**Published:** 2018-11-13

**Authors:** Lisa Hinz, Stephanie D. Hoekstra, Kyoko Watanabe, Danielle Posthuma, Vivi M. Heine

**Affiliations:** 1 Complex Trait Genetics, Center for Neurogenomics and Cognitive Research, Amsterdam Neuroscience, Vrije Universiteit Amsterdam, Boelelaan 1085, 1081HV Amsterdam, The Netherlands; 2 Clinical Genetics, Amsterdam UMC, Amsterdam Neuroscience, Vrije Universiteit Amsterdam, Amsterdam, The Netherlands; 3 Pediatric Neurology, Emma Children’s Hospital, Amsterdam UMC, Amsterdam Neuroscience, Vrije Universiteit Amsterdam, Amsterdam, The Netherlands

**Keywords:** iPSCs, Isogenic control, Episomal reprogramming, X-chromosome, Rett syndrome

## Abstract

Generation of proper controls is crucial in induced pluripotent stem cell (iPSC) studies. X-chromosomal disorders offer the potential to develop isogenic controls due to random X-chromosomal inactivation (XCI). However, the generation of such lines is currently hampered by skewed X-inactivation in fibroblast lines and X-chromosomal reactivation (XCR) after reprogramming. Here we describe a method to generate a pure iPSC population with respect to the specific inactivated X-chromosome (Xi). We used fibroblasts from Rett patients, who all have a causal mutation in the X-linked MeCP2 gene. Pre-sorting these fibroblasts followed by episomal reprogramming, allowed us to overcome skewness in fibroblast lines and to retain the X-chromosomal state, which was unpredictable with lentiviral reprogramming. This means that fibroblast pre-sorting followed by episomal reprogramming can be used to reliably generate iPSC lines with specified X-chromosomal phenotype such as Rett syndrome.

**Electronic supplementary material:**

The online version of this article (10.1007/s12015-018-9851-8) contains supplementary material, which is available to authorized users.

## Introduction

Induced pluripotent stem cells (iPSCs) have revolutionized the field of disease modelling, as they provide a platform for functional investigation of polygenic disorders and diseases of unknown genetic cause that are hard to model in traditional transgenic models. While patient material has given much insight into disease pathology, iPSCs provide the possibility to study important developmental periods of disease onset and progression in specific, human cells. Although iPSC technology opened new possibilities to generate in vitro disease models, the field is still challenged by issues like the lack of standardization, low sample numbers and availability of proper controls [1, 2]. To generate proper controls that consider genetic background as an important variable, previous study designs have included family members [3, 4] or isogenic controls obtained via gene therapy techniques [5]. However, family members are not genetically identical and differences in single nucleotide polymorphisms (SNPs) are not accounted for even though the latter can contribute to they can contribute to, or in some cases determine, disease phenotypes [6]. Furthermore, gene-editing techniques have been shown to have off-target effects [7]. Therefore, there is still a need for alternative approaches to create well-matched controls in iPSC-based studies.

X-chromosomal disorders form a unique group, as affected female patients have cells with either the mutated or the healthy X-chromosome activated in a mosaic expression pattern. This expression provides the opportunity of developing isogenic lines, in which the X-chromosome carrying the mutation is activated in the affected lines and the healthy chromosome is active in the control lines. However, this approach is challenged, as previous studies have obtained mixed lines due to X-chromosomal reactivation (XCR) after reprogramming with lentiviruses [8]. Reprogramming produces iPSC products that either show conserved X-chromosomal inactivation (XCI) or XCR followed by a mosaic or a skewed XCI, indicating that XCI stability during iPSC generation is inconsistent [9, 10]. Different reprogramming methods could be the source of different outcomes in the inactivated X-chromosome (Xi) [11–14]. Therefore, identification of a reprogramming method that retains the XCI could lead to the generation of unmixed lines. Unfortunately, depending on the area from where the sample was taken, skin biopsies of patients may contain both affected and healthy fibroblasts in different rates. Furthermore, it has been shown that culturing and maintenance of fibroblasts can lead to X-chromosomal skewing, which will have an additional effect on the cell ratio [10]. This will lead to complications when attempting to generate unmixed isogenic lines, especially when ratios are heavily skewed. In the current study we offer a solution to overcome skewness of fibroblast lines and XCR by using a reprogramming protocol that consists of fibroblast pre-sorting and a reprogramming method that does not change Xi.

The aim of the current study was to develop an efficient method to obtain isogenic iPSC lines for X-chromosomal disorders. We focus on Rett Syndrome (RTT), a neurodevelopmental disorder that is often associated with mutations in the X-chromosomal Methyl CpG Binding Protein 2 gene (MECP2). These mutations mainly affect females who show a mosaic expression of mutated MECP2. Here we compared a classic lentiviral reprogramming method [15] with an episomal version [16], on mixed and pre-sorted RTT patient fibroblast lines. We show that the iPSCs generated via episomal reprogramming (EiPSCs) did not show XCR of the initial Xi, allowing selection of pure isogenic lines from pre-sorted fibroblasts for functional investigation. On the other hand, iPSCs generated with lentiviruses (ViPSCs) showed a mixed population of MeCP2 expressing cells, even when donor lines all showed XCI of same X-chromosome, thereby hampering the production of pure lines. Based on these results, fibroblast pre-sorting combined with episomal reprogramming appears capable of generating isogenic disease and healthy control lines for iPSC-based studies of X-linked disorders. This could reduce variability and therefore aid discovery of new disease mechanisms and specific targets for therapy.

## Materials and Methods

The investigation of differences in XCR after reprogramming was performed on Rett Syndrome patient cells. We reprogrammed RTT patient fibroblasts via two different reprogramming methods and compared the generated iPSCs. This comparison was done by several assays as immunocytochemistry and RNA-Seq. The generated iPSCs were then differentiated towards all three germ layers via Embryoid Body formation to confirm their pluripotency. Furthermore, neurons were generated from iPSC lines to investigate XCI. All experiments were exempt from approval of Medical Ethical Toetsingscommissie (METC), Institutional Review Board of the VU medical centre.

### Cell Culture

Fibroblasts from three female Rett syndrome patients were obtained from the *Cell lines and DNA bank of Rett Syndrome, X-linked mental retardation and other genetic disease*, member of the Telethon Network of Genetic Biobanks. Each fibroblast line carried a different mutation: Deletion within Exon 3 and 4 of *MECP2*, nonsense mutation p.R255X and p.R270X mutation (here referred to as RTT-FB DEL, RTT-FB R255X and RTT-FB R270X).

Cells were expanded in Fibroblast medium (DMEM-F12, 20% FBS, 1%NEAA, 1%Pen/Strep, 50 μM β-Mercaptoethanol). To generate pure Rett fibroblast lines (RETT-FB DEL MUT) and isogenic controls (RETT-FB DEL CTR) cells were dissociated and single cells were seeded in 96-well plates. After expansion, fibroblasts were tested for MeCP2 expression by PCR and immunocytochemistry.

### Reprogramming

The viral reprogramming was performed using a lentiviral construct containing the classical reprogramming factors *OCT4*, *SOX2*, *KLF4* and *C-MYC* [15].

Episomal reprogramming was achieved as described before with small adjustments [16]. Fibroblasts were dissociated from cell culture plates with Trypsin-EDTA (0,05%) (ThermoFisher). To prepare one well of a 6-well plate, 4 × 10^5^ cells were collected and centrifuged for 5 min at 1200 rpm. Pellet was washed once with PBS and re-suspended in 400 μl Gene Pulser® Electroporation Buffer Reagent (BioRad) containing 23.3 μg of each episomal plasmid (Addgene, Plasmid #27078, #27080, #27076). Cell-plasmid suspension was electroporated in electroporation cuvettes with Gene Pulser II (BioRad). Three pulses of 1.6 kV, with a capacitance of 3 μF and a resistance of 400 Ω were applied. Cells were left to recover overnight in Fibroblast medium without antibiotics but with 10 μM ROCK-inhibitor (Y-27632) in Geltrex®-coated well of a 6 well plate. The next day, medium was changed to Fibroblast medium with antibiotics.

When reprogrammed fibroblasts reached 60–70% confluence, medium was changed to TeSR™-E7™ (STEMCELL) and refreshed daily. Colonies were picked manually after 21–28 days and maintained in TeSR™-E8™ (STEMCELL) on Vitronectin XF™ (STEMCELL) coated 6 well plates [17].

### Embryoid Body Formation and Neuronal Differentiation

To generate Embryoid Bodies (EBs) and test the ability of iPSC lines to form all three germ layers, one well of a 6-well plate of EiPSCs and ViPSCs were dissociated from Vitronectin XF™ (STEMCELL) coated plates with Gentle Cell Dissociation Reagent (STEMCELL). After 5 min incubation Gentle Cell Dissociation Reagent was aspirated and TeSR™-E8™ with 10 μM ROCK-inhibitor was added. Cells were detached by tapping them off the plate and transferred into an anti-adhesive Poly(2-hydroxyethyl methacrylate) (Sigma-Aldrich) coated well of a 6-well plate. Next day, EB-formation was checked and half of the medium (TeSR™-E8™ with 10 μM ROCK-inhibitor) was changed. Floating EBs were cultured for 10 days and half of medium was replaced every other day with TeSR™-E8™. After 10 days EBs were plated on Geltrex®-coated coverslips in TeSR™-E8™ and kept in culture for another 3 days. Afterwards, EBs were fixated and stained for germ layer markers. Neuronal differentiation was performed as described before [18]. Generated neurons were fixated and immunocytochemically stained for neuronal marker and MeCP2.

### Immunocytochemistry

For immunocytochemistry, cells were plated on Vitronectin XF™-coated coverslips and fixated with 4% paraformaldehyde for 10 min followed by PBS wash (3×). Blocking and permeabilization were performed by a one-hour incubation in blocking buffer (PBS, 5% normal goat serum (Gibco®), 0.1% bovine serum albumin (Sigma-Aldrich), 0.3% Triton X-100 (Sigma-Aldrich)). Primary antibodies for MeCP2 (D4F3, CellSignaling, 1:200, rabbit), H3K27me3 (C36B11, CellSignaling, 1:1600, rabbit), OCT4 (C-10, Santa Cruz, 1:1000, mouse), SSEA4 (Developmental Studies Hybridoma Bank, 1:50, mouse), TRA1–60 (Santa Cruz, 1:200, mouse), TRA1–81 (Millipore, 1:250, mouse), SOX2 (Millipore, 1:1000, rabbit), β-III Tubulin (R&D Systems, 1:1000, mouse), α-Fetoprotein (R&D Systems, 1:1000, mouse), α-Smooth Muscle Actin (SMA) (Progene, 1:1000, mouse), MAP2 (Abcam, 1:500, chicken), SMI312 (Eurogentec, 1:1000, mouse) and VGLUT2 (SYnaptic SYstems, 1:1000, rabbit) were diluted in blocking buffer and incubated for 1 h at room temperature followed by an overnight incubation at 4 °C. After PBS wash, secondary antibodies Alexa Fluor® 488 (ThermoFisher, 1:1000, mouse) and Alexa Fluor® 594 (ThermoFisher, 1:1000, mouse or rabbit) diluted in blocking buffer were applied to the cells for 1–2 h at room temperature. DAPI staining was performed in PBS for 5 min and coverslips were mounted on glass slides with Fluoromount™ (Sigma-Aldrich).

### RT-PCR

Total RNA from EiPSCs and ViPSCs was collected after ten passages (P10) and isolated by standard TRIzol®-chloroform isolation and iso-propanol precipitation. cDNA was derived from the collected mRNA by using SuperScript IV Reverse Transcriptase (SSIV) (ThermoFisher). For RT-PCR 1 μg of RNA and Random Hexamer Primers were used and incubated with SSIV, 0,1 M Dithiothreitol (DTT) and 40 U/μl RNaseOUT (ThermoFisher) for 10 min at 23 °C, 10 min at 50 °C and 10 min at 80 °C. Afterwards samples were stored at −20 °C until further processed. PCR analysis was performed with different primer sets (Tab. S1) with Phire Hot Start II DNA Polymerase (ThermoFisher). To perform PCR, a mastermix containing Phire Buffer (ThermoFisher), dNTPs (10 mM) (ThermoFisher) and Phire Hot Start II DNA Polymerase was mixed and divided over different PCR tubes. 1 μl of each primer (10 μM) and 1 μl cDNA was added to the tubes and carefully mixed. Samples were placed in thermocycler (Applied Biosystems™ 2720 Thermal Cycler) and PCR program was initiated (Tab. S2).

### RNA Sequencing

Total RNA was isolated from iPSCs during passaging using TRIzol®-chloroform isolation as described above. RNA quality was evaluated by calculating the RNA integrity number (RIN; Agilent Technologies) using an Agilent 2200 TapeStation system (tape D5000). Samples with RIN ≥ 8 were selected and used to prepare libraries for RNA sequencing using the TruSeq mRNA sequencing kit (Illumina) according to manufacturer’s recommendations. Loading concentration of 100 to 200 ng RNA were used for the library preparation. Briefly, mRNA was purified using poly-T oligo magnetic beads and then fragmented. Reverse transcription was performed using SuperScript IV Reverse Transcriptase (SSIV) (ThermoFisher) and First Strand Synthesis Act D Mix (Illumina) followed by production of the second strand with incorporation of dUTP using Second Strand Marking Master Mix (Illumina) and purification of samples. This was followed by A-tailing and adaptor ligation (Ligation Mix and Stop Ligation Buffer, Illumina). Samples were washed using the AMPure XP Beads (Beckman Coulter) DNA fragments were enriched using PCR Primer Cocktail and PCR Master Mix for 15 cycles (Illumina) and a last purifications step was performed. Library quality control was performed using the Agilent Technologies 2100 Bioanalyzer where after samples were sequenced with the Illumina HiSeq 4000 (50 base pair single read).

Quality control was performed using FastQC software and sequencing reads aligned to Human Genome hg38 by STAR v2.5.3a [19]. Expression value was quantified as, expected count and transcripts per million (TPM), using RSEM v1.3.0 [20] with GENCODE v25 gene annotation (http://www.gencodegenes.org/) which contains 63,299 genes.

### PluriTest

PluriTest was performed for samples by submitting fastq file to the PluriTest server (https://pluritest.org/) [21].

### Statistics

A T-Test was performed to compare the expression levels of *XIST* between EiPSCs and ViPSCs using R programming language software.

## Results

### X-Chromosomal State after Using Different Reprogramming Methods

**Fig. 1:**
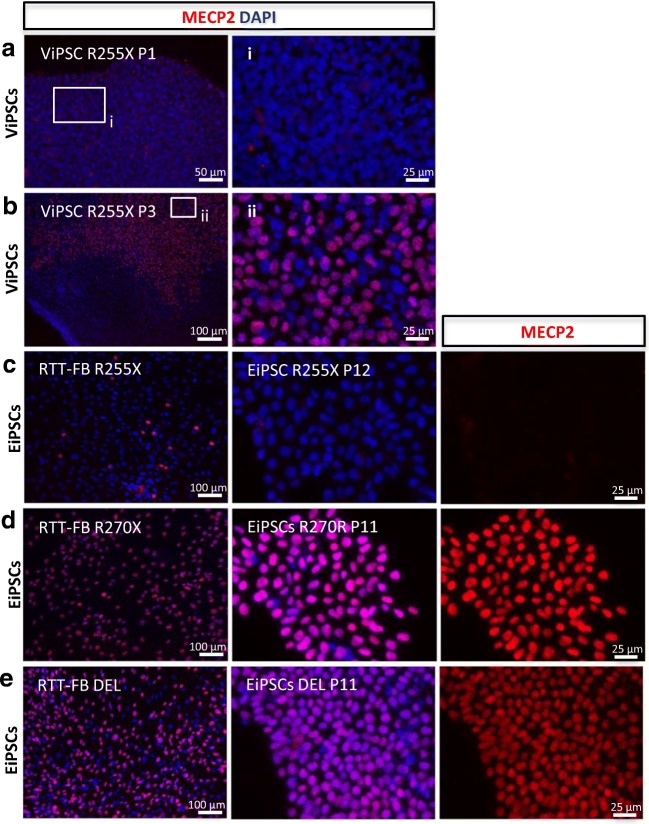
**MeCP2 expression after reprogramming.** Immunocytochemistry for MeCP2 of **a** patient R255X line after viral reprogramming at ViPSC passage 1 (P1) and **b** passage 3 (P3); **c** patient RTT-FB R255X **d** patient RTT-FB R270X lines and **e** patient RTT-FB DEL at fibroblast stage and after episomal reprogramming (the EiPSC stage), respectively, at passage 12 and 11 (P12 + P11)

To find a reprogramming method in which the XCI of the generated iPSCs can be controlled, we created iPSC lines from three Rett patients with MeCP2 deficiencies using both, the classic lentiviral and the non-integrative episomal reprogramming method. We reprogrammed skin fibroblasts from a Rett patient carrying the R255X mutation (RTT-FB R255X), whose fibroblast line was mainly negative for MeCP2 expression (p5%:n95%). After the lentiviral reprogramming, we selected 9 colonies for further passage and characterization (Fig. S1). All lentiviral generated iPSC lines (ViPSCs R255X) showed pure MeCP2-negative colonies in P1–2 (Fig. 1a). After P3, MeCP2-positive cells appeared in these initially negative iPSC lines (Fig. 1b). Beyond passages 4–9, 5 out of 9 iPSC lines consisted of mixed populations of MeCP2-positive and –negative cells (Fig. 1b).

After episomal reprogramming of RTT-FB R255X, we selected and characterized 10 colonies. In contrast to lentiviral reprogramming, colonies generated via episomal reprogramming (EiPSCs R255X) were all MeCP2-negative at every characterized passage (Fig. 1c). To further confirm that episomal reprogramming can generate iPSC populations and maintain the Xi present at the fibroblast stage, we performed the same reprogramming procedures on two more Rett patient fibroblast lines. We episomally reprogrammed fibroblast lines in which the majority of cells were MeCP2-positive (RTT-FB R270X, p97%:n3% and RETT-FB DEL, p70%:n30%). After episomal reprogramming, all 10 iPSC lines from RTT-FB R270X (EiPSC R270X) and all 10 iPSC lines from RETT-FB DEL (EiPSC DEL) resulted in solely MeCP2-positive iPSC colonies (Fig. 1d+e). All cells in these colonies for both patients remained positive for MeCP2 expression after more than 10 passages (Fig. 1d+e).

### Single Fibroblast Sorting to Generate Mutated Cell Lines and Isogenic Controls

Selection of specific cell populations from mixed iPSC lines is still challenging. While episomal reprogramming methods can potentially generate an iPSC line with the same Xi, fibroblast lines with low percentages of cells containing the X-linked mutation or the X-linked healthy genetic variant might not generate iPSC lines of both identities (Fig. 1c-e). To overcome these issues, we investigated whether pre-sorting of fibroblasts in combination with episomal reprogramming, would generate pure iPSC lines with the identical Xi.

**Fig. 2:**
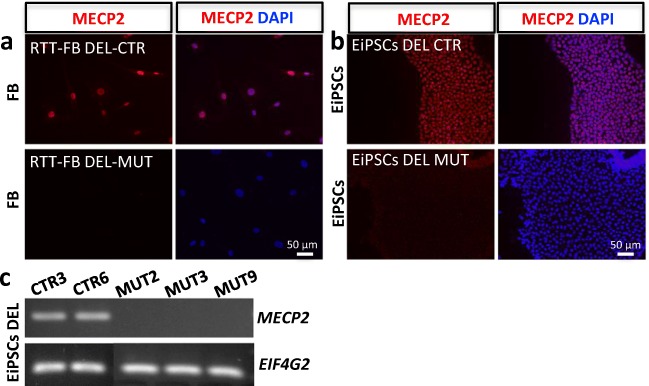
**Generation of RTT and isogenic control cell line.** Immunocytochemistry for MeCP2 of (**a**) manually-sorted RTT patient fibroblasts. Into MeCP2-positive (upper panels) and –negative (lower panels) cells, and (**b**) the EiPSC-derived lines. **c** PCR analysis of MeCP2 expression of cell lines EiPSC DEL CTR and EiPSC DEL MUT

To create a fibroblast line from RTT-FB DEL with a homogeneous maternal or paternal X-chromosomal state, we plated single cells into a 96-well plate. About 30% of these cells attached and could be passaged further. Fibroblast clones were characterized as pure MeCP2-positive (RTT-FB DEL CTR, Fig. 2a top), pure MeCP2-negative (RTT-FB DEL MUT, Fig. 2a bottom) or mixed population (not shown), using immunocytochemistry and PCR analysis (Fig. 2c). Episomal reprogramming was performed with RTT-FB DEL CTR and RTT-FB DEL MUT fibroblast lines to investigate if their Xi state could be maintained. We picked 10 colonies per fibroblast line and characterized these for MeCP2 expression. PCR analysis confirmed that all picked colonies for the MUT fibroblast line showed a pure population of MeCP2-negative cells up to P14 (Fig. 2c), which is in accordance with the MeCP2 state of the reprogrammed fibroblast line. Furthermore, all iPSC lines derived from the CTR fibroblast line showed a clear positive result for MeCP2 in PCR analysis (Fig. 2c). While PCR analysis does not exclude the presence of MeCP2-negative cells, immunocytochemical inspection for MeCP2 of selected samples showed pure populations of MeCP2-positive cells (Fig. 2b). Therefore, we were able to generate exclusively MeCP2-positive EiPSC colonies from RTT-FB DEL CTR and entirely MeCP2-negative colonies from RTT-FB DEL MUT (Fig. 2b).

### Chromatin State during Early iPSC Passages

**Fig. 3:**
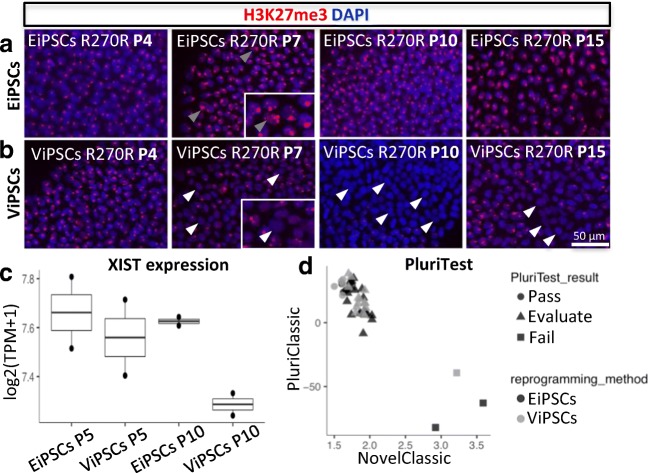
**H3K27me3 condensation and*XIST*expression in early ViPSC and EiPSC passage lines.** Immunocytochemistry for H3K27me3 of control iPSC lines at passages P4, P7, P10, P15 generated via **a** episomal reprogramming and **b** viral reprogramming. **c** RNA-Seq analysis of XIST for episomal and viral reprogrammed lines at passage P5 and P10 (two-sided t-test, *n* = 2, *p* = 0,04, data is represented at mean). PluriTest scores for all EiPSCs and ViPSCs

In previous studies, the X-chromosomal state of cells was investigated by different approaches. Immunocytochemistry for H3K27me3 provides one option for visualizing Xi. An acquisition of this histone methylation is known to support the XCI and can be observed as a high conjugated spot in the nucleus [22–24]. To confirm that the viral and episomal reprogramming methods result in different X-chromosomal states in obtained iPSC lines, we characterized the condensation of H3K27me3 by immunostaining. H3K27me3 staining analysis was performed at different passages in two different clones of EiPSC R270X (EiPSC R270X_1 and EiPSC R270X_2) and ViPSC R270X (ViPSC R270X_1 and ViPSC R270X_2). The iPSCs from both reprogramming methods showed a condensed spot of H3K27me3 labeling during the first passages (P1–4) (Fig. 3a and b), which changed after higher passage numbers. At P7, single groups of cell nuclei negative for H3K27me3 appeared in ViPSC clones (Fig. 3b) and increased in cell numbers during further passages. At P10, ViPSC colonies were partly or totally negative for H3K27me3 (Fig. 3b) and stayed mixed up to P15 (Fig. 3b). In contrast, all generated EiPSC lines, partly showing scattered H3K27me3 condensation in some nuclei (Fig. 3a), were positive for H3K27me3 at all passages up to P15 (Fig. 3a).

Furthermore, expression of X-inactive specific transcript (XIST) plays a key role in X-chromosomal inactivation [25–27]. The expression of XIST in ViPSCs and EiPSCs at P4 and P10 was studied using RNA-Seq analysis (Fig. 3c). At P4, both types of iPSCs show a relatively high expression of XIST RNA. When directly compared no significant difference in XIST expression levels between ViPSCs and EiPSCs was observed (n.s., two-sided t-test), further confirming the presence of XCI at P4 using either methods. However, at P10 ViPSCs showed significantly decreased XIST expression, when compared expression levels of EiPSCs (*p* value = 0.04, two-sided t-test), indicating XCR in ViPSCs. In contrast EiPSCs showed a stable XIST expression throughout passages (Fig. 3c). To confirm the stability of the XCI in EiPSCs during long term culture, we have differentiated EiPSCs into neuron according to the protocol described by Nadadhur et al. [18] (Fig. S2). All neurons show that the XCI of the original fibroblast lines was retained until a mature neural stage. Furthermore, this confirms the potency of the EiPSCs to differentiate into mature neurons despite the lack of XCR.

### Pluripotent State of iPSCs after Different Reprogramming Methods

**Fig. 4:**
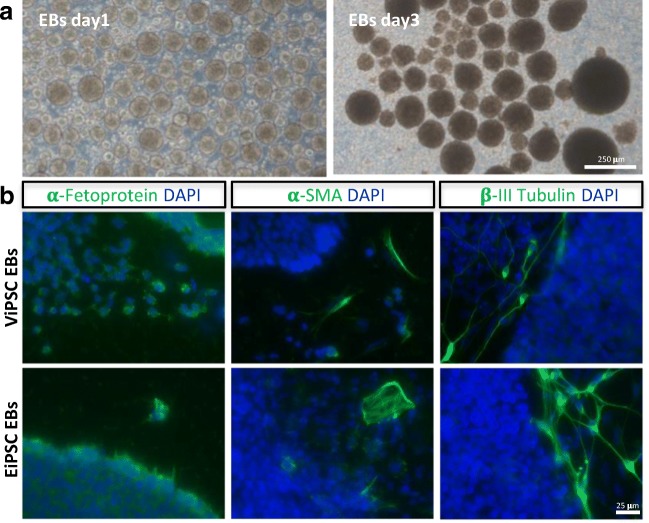
**Embryoid Body formation**. **a** Brightfield images of day 1 and 3 of embryoid bodies formed from iPSCs. **b** Immunocytochemistry of both ViPSC- derived EBs (upper panels) and EiPSC- derived EBs (lower panels) for all three germ layers: (i) α-Fetoprotein (endoderm), (ii) α-SMA (mesoderm) and (iii) β-III Tubulin (ectoderm)

To investigate whether the reprogramming methods differently affected the pluripotency state of the iPSC lines, we performed RNA-Seq followed by Pluri-Test analysis [21]. ViPSCs and EiPSCs equally passed the PluriTest (ViPSCs passed:19,2% evaluate:80,8% EiPSCs passed:17,6% evaluate: 82,4%) and did not cluster separately (Fig. 3d). Pluripotency of iPSC lines was further evaluated by germ layer formation, which showed expression of mesodermal, endodermal and ectodermal markers in the iPSC derived from both methods (Fig. 4). This indicates that both ViPSCs and EiPSCs reached the pluripotency state making them suitable for follow-up studies.

## Discussion

Here we showed that episomal and lentiviral reprogramming affects XCI differently. This observation could aid the generation of iPSC lines from patients with an X-chromosomal disorder with the uniform inactivation of chromosomes carrying either the mutated or healthy gene (i.e. isogenic controls). Based on X-linked expression of MeCP2, we showed that XCI at the fibroblast stage remains stable during episomal reprogramming, but becomes unpredictable during lentiviral reprogramming. Furthermore, based on histone condensation marker H3K27me3 and XIST expression we showed XCR in a subset of cells generated via viral reprogramming method. This is in accordance with the mixed population of MeCP2-positive and –negative cells seen in colonies of ViPSC R255X, which appeared from P3 onwards (Fig. 1b). In contrast, all passages of EiPSCs showed stable MeCP2-expression consistent with their donor fibroblasts (Fig. 1c-e). The stable MeCP2 expression shown in EiPSCs, is an indication that episomal reprogramming in pre-sorted fibroblast lines can be a reliable, efficient and less cost-intensive method to generate affected iPSCs and isogenic controls for the investigation of X-chromosomal-linked disorders, such as Rett syndrome.

### Robust Generation of Isogenic Controls by Episomal Reprogramming

Lentiviral reprogramming of the mostly MeCP2-negative fibroblast line RTT-FB R255X led to XCR, resulting in a mixed population of MeCP2-positive and –negative iPSCs. However, this was not the case for every ViPSC R255X clone. Four out of nine lines stayed negative for MeCP2, while the remaining five lines showed different ratios of MeCP2-positive and -negative cells. Even though purely negative lines were generated by lentiviral reprogramming, none of our lines was entirely positive for MeCP2. Therefore, as described before [8, 11, 14], lentiviral reprogramming is unpredictable and can lead to a random XCI after reprogramming and during iPSC maintenance.

In contrast, episomal reprogramming of mainly MeCP2-negative fibroblast line RTT-FB R255X only generated EiPSC lines with MeCP2-negative cells, which did not show MeCP2 expression throughout all analysed passages (Fig. 1c). This suggests that episomal reprogramming does not reactivate the silenced X-chromosome. We confirmed this by the episomal reprogramming of the mainly MeCP2-positive fibroblast line RTT-FB DEL and RTT-FB R270X. All iPSC lines derived from the latter fibroblasts were MeCP2-positive and maintained their MeCP2 identity for over 12 passages (Fig. 1d+e). Nevertheless, XCR could have taken place, as the affected cells would still have shown a MeCP2 positive staining. However, RNA sequencing showed stable levels of XIST expression throughout several passages, suggesting no XCR took place in these cells (Fig. 1c). This implies a stable Xi within the EiPSCs after episomal reprogramming, which depends on the XCI of the used fibroblasts. This was further confirmed by long-term differentiation towards neurons, where all cells retained the original MeCP2 expression of the fibroblast line they were derived from (Fig. S2).

To generate purely MeCP2- positive and –negative iPSC lines from a mixed fibroblast line, single-cell-plated fibroblasts were expanded and characterized for their MeCP2-state. The cloned fibroblast lines were used to generate pure MeCP2-mutant and isogenic control lines of the same patient. Previous studies had to perform several rounds of reprogramming in order to generate isogenic iPSC lines due to skewing issues [10, 11, 14]. Therefore, our approach involving single-cell-plating fibroblasts and opting for episomal reprogramming, can be a more efficient and less cost-intensive method.

### Pluripotency Presents with Different X-Chromosomal States

In vivo, the naïve pluripotent stem cell state in female cells is referred to a state in which both X-chromosomes are active [28]. However, earlier studies indicated that in vitro*,* XCI is induced by passaging, maintenance and early differentiation of pluripotent stem cells into somatic cells [29]. While all of our lines show the standardized pluripotency markers (Fig. S1), this could suggest that ViPSCs are more naïve than EiPSCs. This could not be confirmed by the characterisation of EiPSC and ViPSCs using RNA seq. The results of the Pluri-Test showed no separate clustering of the different iPSCs. Furthermore, Pluri-Test in combination with classic characterization analysis indicated that both ViPSC and EiPSC lines are pluripotent.

### iPSC Reprogramming Induces Poised XCI

The absence of H3K27me3 shows XCR in the lentiviral reprogramming method. As we observed disappearance of H3K27me3 in nuclei of the ViPSCs from P7 onwards but not in EiPSCs, we conclude that the XCR only establishes beyond early passaging. This difference became more obvious after P10, in which we observed big parts or entire colonies of ViPSCs to be negative for H3K27me3. To ensure XCR was not delayed but lost in EiPSCs we cultured both lines up to P15. No H3K27me3 negative nuclei appeared in EiPSCs (Fig. 3a), while we clearly observed them in ViPSCs (Fig. 3b). Although EiPSCs were positive for H3K27me3 at all passages, clear chromosomal condensation was sometimes partly lost, thereby creating a more scattered signal (Fig. 3a). A possible explanation for this is that episomal reprogramming led to partial reactivation of both X-chromosomes, but due to epigenetic memory and skewing, the formerly inactivated chromosome is again inactivated [10]. However, this seems implausible since we did not observe MeCP2 expression at any stage during episomal reprogramming in line EiPSC-R255X, suggesting that the X-chromosome was inactive throughout cultivation. Another possible explanation is, that even though the chromosome seems to be partly unwound, it remains inactive due to the expression of other factors involved in Xi, such as XIST [30]. RNA-Seq revealed a decreased XIST expression at P10 in ViPSCs, which coincides with the loss of H3K27me3 condensation. The same effect was not observed in EiPSCs, as XIST expression remained stable. These differences in XIST expression might explain why viral reprogramming leads to reactivation of the silenced X-chromosome, while episomal reprogramming does not [10, 12, 31]. Taken together, the absence of H3K27me3 condensation in ViPSCs and decreased XIST expression suggests XCR. As no changes in expression of XIST or H3K27me3 were seen in EiPSCs, in combination with uniform MeCP2 stainings, we conclude episomal reprogramming does not affect the X-chromosomal state of donor cells.

### Proper Controls in iPSC-Based Models for X-Linked Disorders

There is currently more awareness that genetic background could contribute to disease phenotypes in iPSC-based studies and should be accounted for [1]. Especially when genes of interest, such as MeCP2, interact with other genes and different pathways, the generation of isogenic controls can be very important. Absence of isogenic controls weakens conclusions regarding causal effect of the mutation alone on observed phenotype. Our approach to generate proper controls, is not only useful for development of in vitro models of RTT, but also for other dominant X-linked diseases like Fragile X or Coffin-Lowry syndrome [32–34].

Taken together, the presented method of generating disease and control lines from patients with dominant X-chromosomal disorders facilitates in vitro iPSC-based disease modelling, which can be hindered by high variability. It offers an efficient and inexpensive approach to generate iPSC populations with either only the mutated or the healthy X-chromosomes. This approach, in combination with other recent advances in the stem cell field to reduce variability, such as standardization and automation, are of utmost importance to generate valid disease models.

## Electronic supplementary material

ESM 1 (PPTX 63 kb)

Fig. S1 **Representative images of iPSC characterisation**. (A) Reprogramming of female fibroblast. (B) Immunocytochemistry of EiPSCs pluripotency markers Sox2, SSEA4, Tra 1–60 and Tra 1–81. (C) PCR results of iPSCs for pluripotency marker Oct3/4, Sox2, Nanog, C-Myc, TDGF-1, UTF-1 and DNMT3B. (D) Representative alkaline phosphatase staining of an EiPSC colony and individual EiPSCs in one well of a 24WP. (E) Immunocytochemistry of β-III tubulin, α-fetoprotein and smooth muscle actin in embryoid bodies (EBs). (PPTX 25711 kb)

Fig. S2 **Neuronal differentiation of Rett EiPSCs and isogenic controls**. Representative immunocytochemistry of differentiated neuronal cells for MAP2, SMI312, Vglut2 and DAPI (A). Immunocytochemistry of differentiated neurons from EiPSCs_DEL CTR (B) and EiPSCs_DEL MUT (C) for MeCP2. (PPTX 7431 kb)

Fig. S3 **Negative controls for immunocytochemistry**. H3K27me3 staining of male (upper) and female (lower) fibroblasts to ensure specific binding of Xi condensation (A). Only secondary antibody staining of iPSCs for Alexa-fluor goat anti mouse 594 and 488 (left) and Alexa-fluor goat anti rabbit 594 and 488 (right)(B). (PPTX 6162 kb)
