# Supplementary material for: Generation of Isogenic Controls for In Vitro Disease Modelling of X-Chromosomal Disorders
Source: Stem Cell Rev. 2018 Nov 13;15(2):276–85. doi: 10.1007/s12015-018-9851-8 (PMC6441401; doi:10.1007/s12015-018-9851-8)
Supplement: Supplementary file 1 — (PPTX 63 kb) [file 12015_2018_9851_MOESM1_ESM.pptx]

## Slide 1
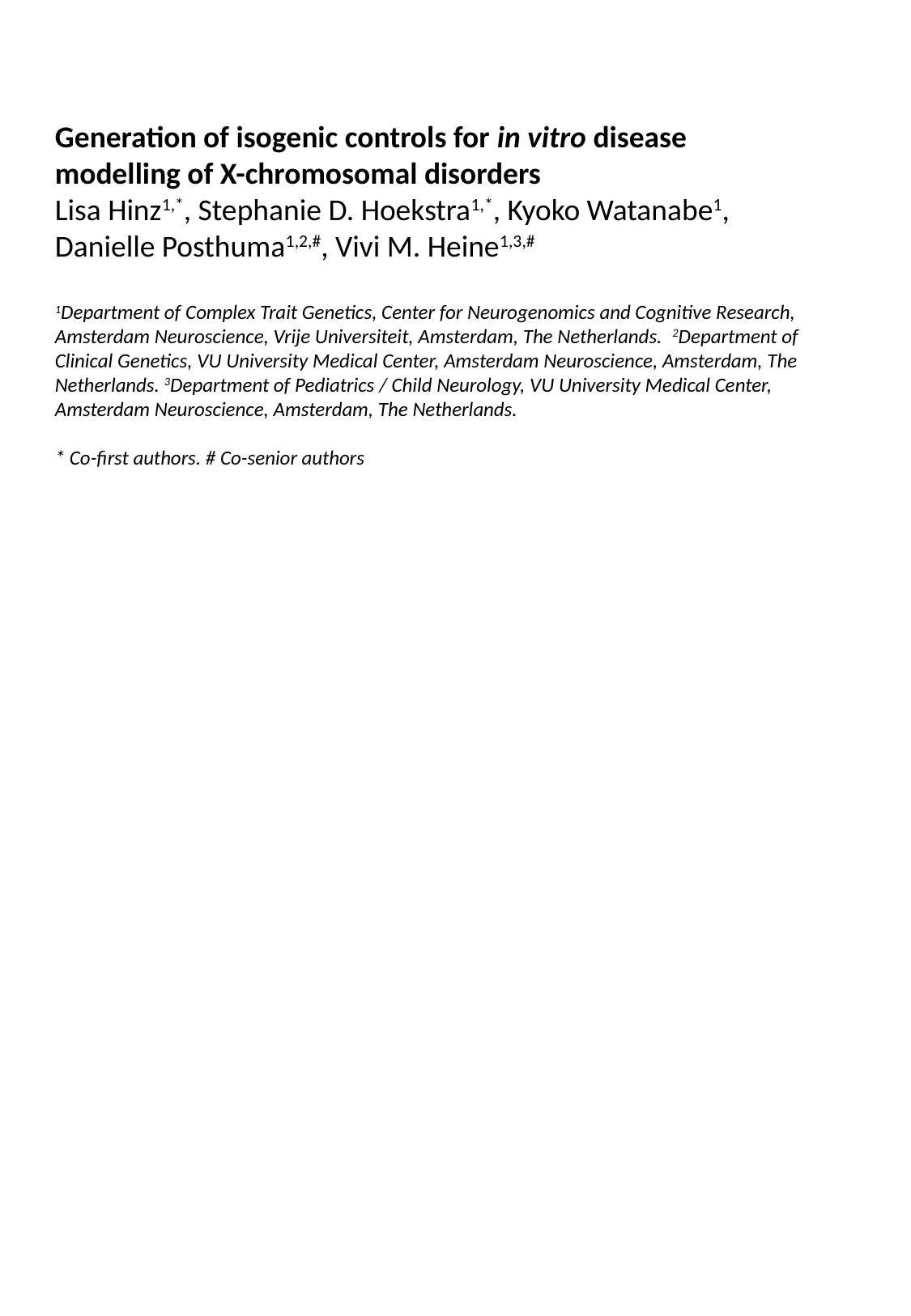

Generation of isogenic controls for in vitro disease modelling of X-chromosomal disorders
Lisa Hinz1,*, Stephanie D. Hoekstra1,*, Kyoko Watanabe1, Danielle Posthuma1,2,#, Vivi M. Heine1,3,#
1Department of Complex Trait Genetics, Center for Neurogenomics and Cognitive Research, Amsterdam Neuroscience, Vrije Universiteit, Amsterdam, The Netherlands. 2Department of Clinical Genetics, VU University Medical Center, Amsterdam Neuroscience, Amsterdam, The Netherlands. 3Department of Pediatrics / Child Neurology, VU University Medical Center, Amsterdam Neuroscience, Amsterdam, The Netherlands.
* Co-first authors. # Co-senior authors

## Slide 2
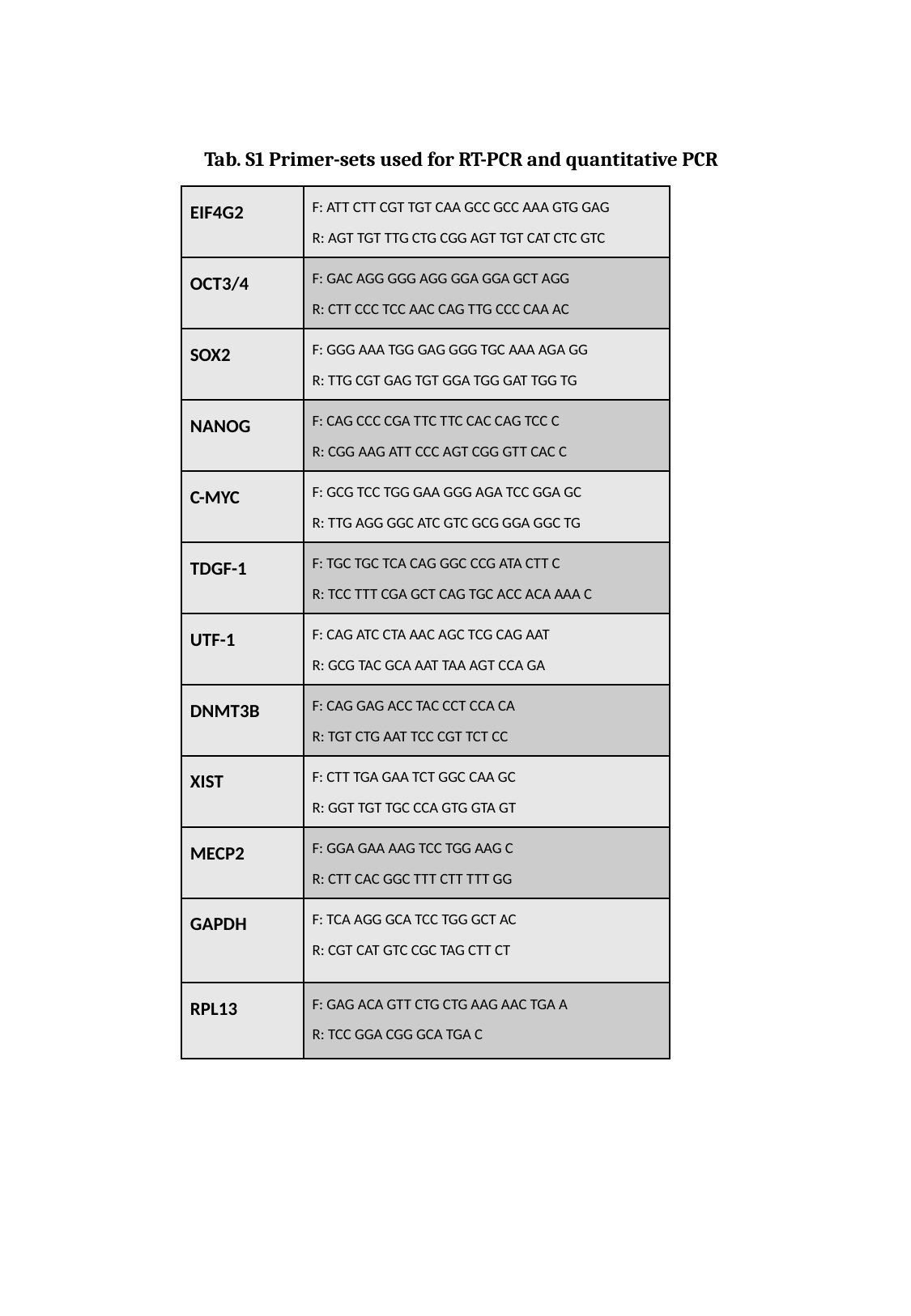

Tab. S1 Primer-sets used for RT-PCR and quantitative PCR
| EIF4G2 | F: ATT CTT CGT TGT CAA GCC GCC AAA GTG GAG R: AGT TGT TTG CTG CGG AGT TGT CAT CTC GTC |
| --- | --- |
| OCT3/4 | F: GAC AGG GGG AGG GGA GGA GCT AGG R: CTT CCC TCC AAC CAG TTG CCC CAA AC |
| SOX2 | F: GGG AAA TGG GAG GGG TGC AAA AGA GG R: TTG CGT GAG TGT GGA TGG GAT TGG TG |
| NANOG | F: CAG CCC CGA TTC TTC CAC CAG TCC C R: CGG AAG ATT CCC AGT CGG GTT CAC C |
| C-MYC | F: GCG TCC TGG GAA GGG AGA TCC GGA GC R: TTG AGG GGC ATC GTC GCG GGA GGC TG |
| TDGF-1 | F: TGC TGC TCA CAG GGC CCG ATA CTT C R: TCC TTT CGA GCT CAG TGC ACC ACA AAA C |
| UTF-1 | F: CAG ATC CTA AAC AGC TCG CAG AAT R: GCG TAC GCA AAT TAA AGT CCA GA |
| DNMT3B | F: CAG GAG ACC TAC CCT CCA CA R: TGT CTG AAT TCC CGT TCT CC |
| XIST | F: CTT TGA GAA TCT GGC CAA GC R: GGT TGT TGC CCA GTG GTA GT |
| MECP2 | F: GGA GAA AAG TCC TGG AAG C R: CTT CAC GGC TTT CTT TTT GG |
| GAPDH | F: TCA AGG GCA TCC TGG GCT AC R: CGT CAT GTC CGC TAG CTT CT |
| RPL13 | F: GAG ACA GTT CTG CTG AAG AAC TGA A R: TCC GGA CGG GCA TGA C |

## Slide 3
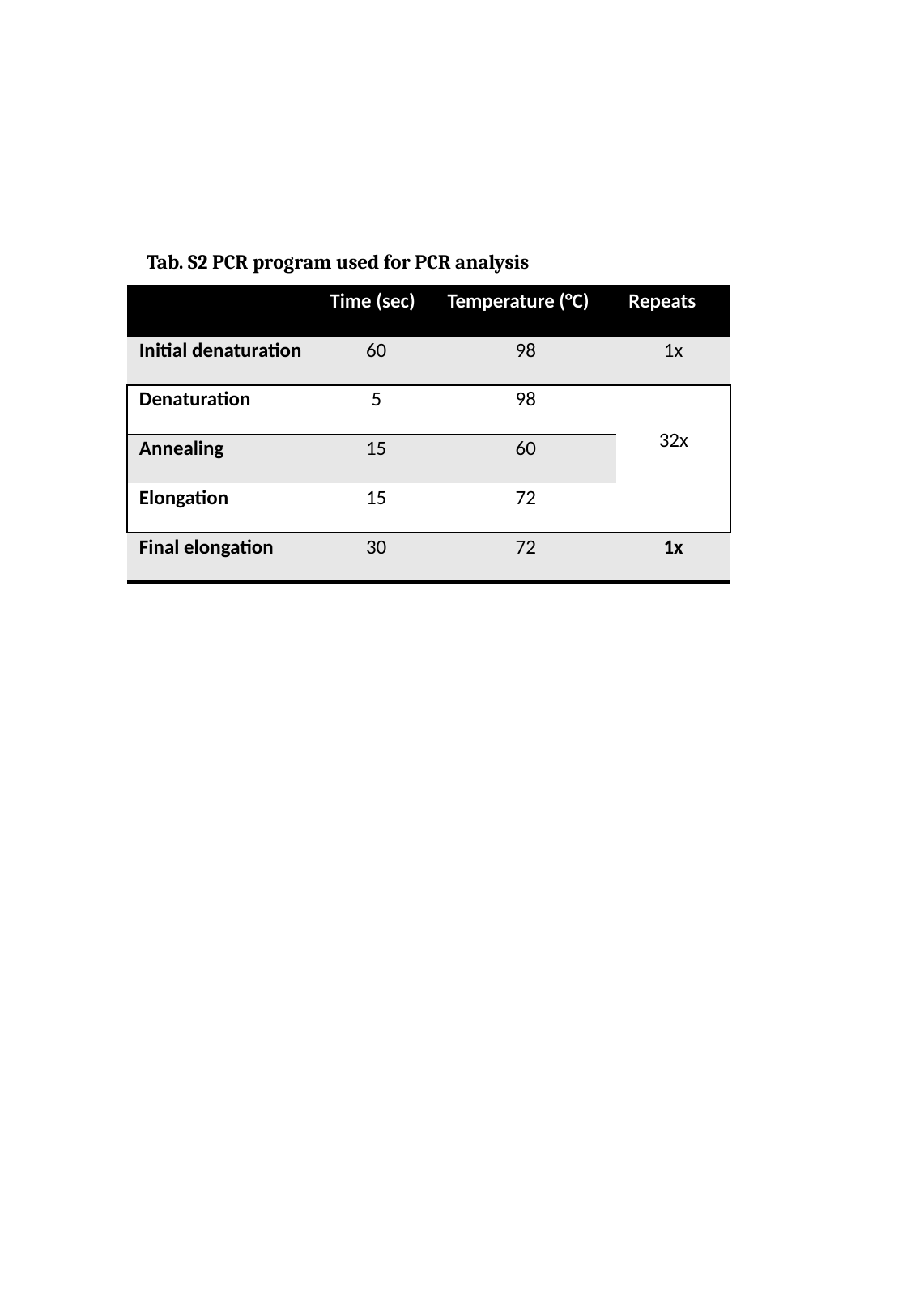

Tab. S2 PCR program used for PCR analysis
| | Time (sec) | Temperature (°C) | Repeats |
| --- | --- | --- | --- |
| Initial denaturation | 60 | 98 | 1x |
| Denaturation | 5 | 98 | 32x |
| Annealing | 15 | 60 | |
| Elongation | 15 | 72 | |
| Final elongation | 30 | 72 | 1x |
