# Supplementary material for: Generation of Isogenic Controls for In Vitro Disease Modelling of X-Chromosomal Disorders
Source: Stem Cell Rev. 2018 Nov 13;15(2):276–85. doi: 10.1007/s12015-018-9851-8 (PMC6441401; doi:10.1007/s12015-018-9851-8)
Supplement: Supplementary file 2 — Representative images of iPSC characterisation. (A) Reprogramming of female fibroblast. (B) Immunocytochemistry of EiPSCs pluripotency markers Sox2, SSEA4, Tra 1–60 and Tra 1–81. (C) PCR results of iPSCs for pluripotency marker Oct3/4, Sox2, Nanog, C-Myc, TDGF-1, UTF-1 and DNMT3B. (D) Representative alkaline phosphatase staining of an EiPSC colony and individual EiPSCs in one well of a 24WP. (E) Immunocytochemistry of β-III tubulin, α-fetoprotein and smooth muscle actin in embryoid bodies (EBs). (PPTX 25711 kb) [file 12015_2018_9851_MOESM2_ESM.pptx]

## Slide 1
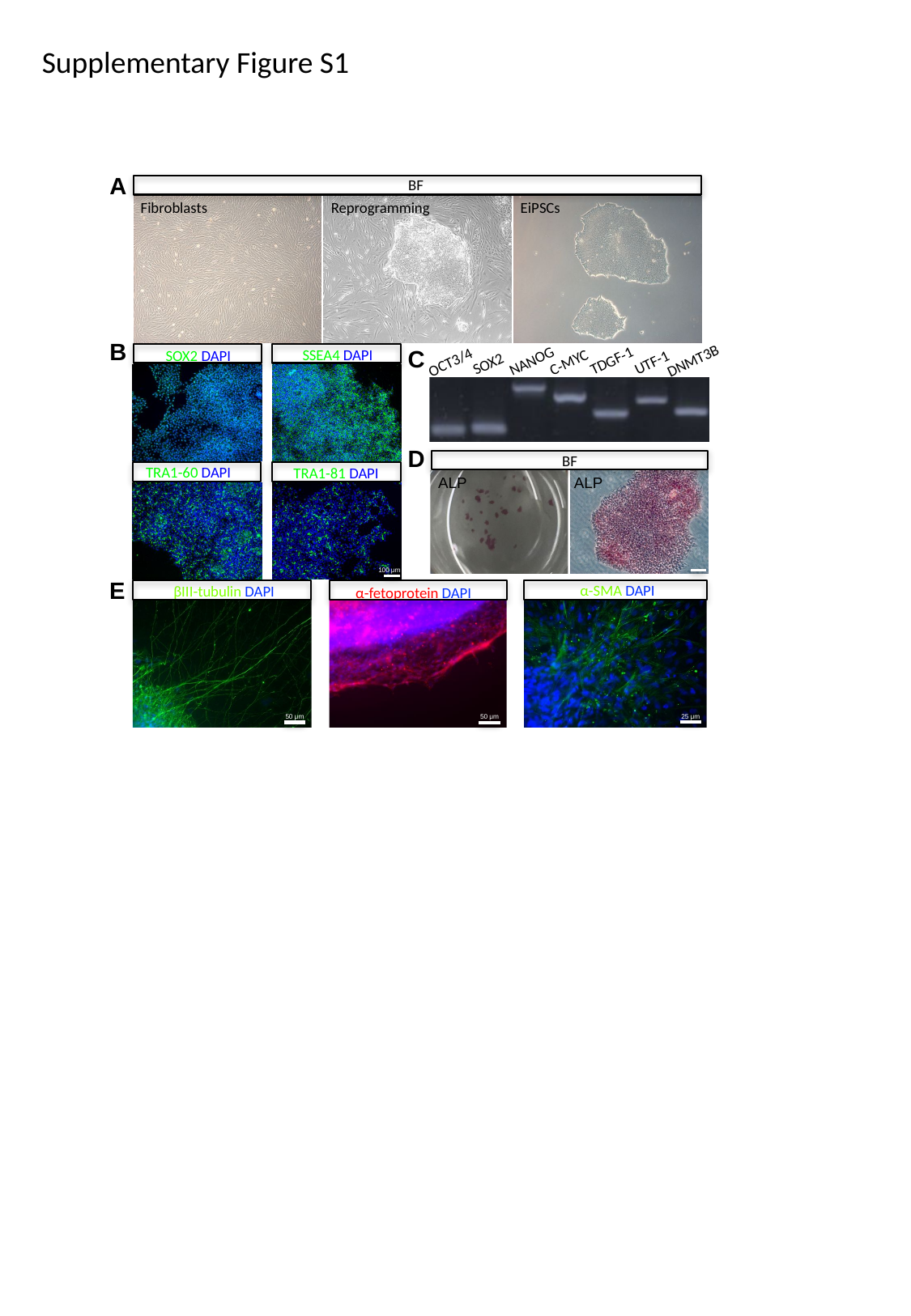

Supplementary Figure S1
A
BF
EiPSCs
Reprogramming
Fibroblasts
B
C
SSEA4 DAPI
SOX2 DAPI
TDGF-1
NANOG
DNMT3B
C-MYC
OCT3/4
UTF-1
SOX2
D
BF
TRA1-60 DAPI
TRA1-81 DAPI
ALP
ALP
100 μm
E
α-SMA DAPI
βIII-tubulin DAPI
α-fetoprotein DAPI
50 μm
50 μm
25 μm
