# Supplementary material for: Generation of Isogenic Controls for In Vitro Disease Modelling of X-Chromosomal Disorders
Source: Stem Cell Rev. 2018 Nov 13;15(2):276–85. doi: 10.1007/s12015-018-9851-8 (PMC6441401; doi:10.1007/s12015-018-9851-8)
Supplement: Supplementary file 3 — Neuronal differentiation of Rett EiPSCs and isogenic controls. Representative immunocytochemistry of differentiated neuronal cells for MAP2, SMI312, Vglut2 and DAPI (A). Immunocytochemistry of differentiated neurons from EiPSCs_DEL CTR (B) and EiPSCs_DEL MUT (C) for MeCP2. (PPTX 7431 kb) [file 12015_2018_9851_MOESM3_ESM.pptx]

## Slide 1
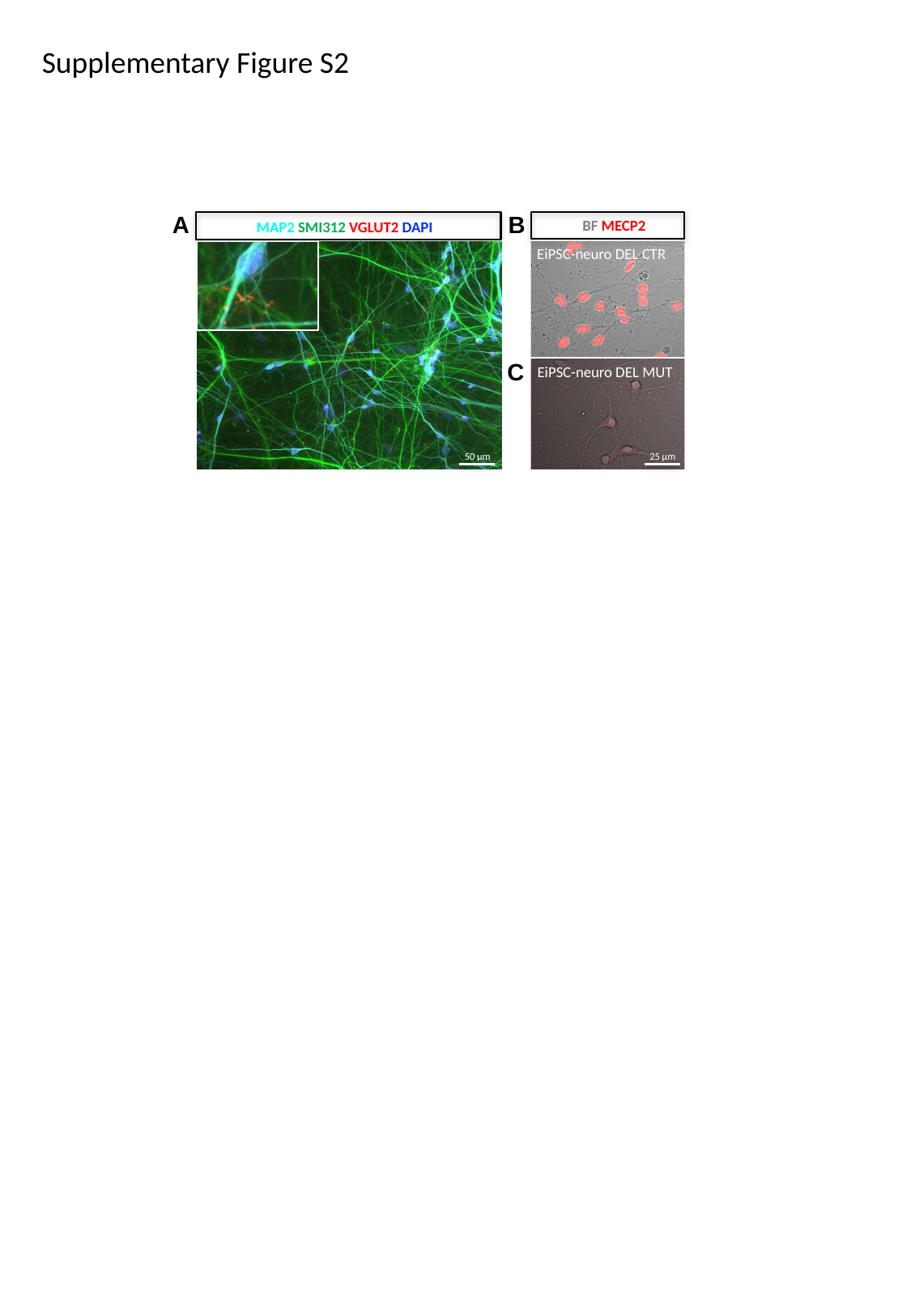

Supplementary Figure S2
A
B
BF MECP2
MAP2 SMI312 VGLUT2 DAPI
EiPSC-neuro DEL CTR
50 μm
C
EiPSC-neuro DEL MUT
25 μm
