# Supplementary material for: Generation of Isogenic Controls for In Vitro Disease Modelling of X-Chromosomal Disorders
Source: Stem Cell Rev. 2018 Nov 13;15(2):276–85. doi: 10.1007/s12015-018-9851-8 (PMC6441401; doi:10.1007/s12015-018-9851-8)
Supplement: Supplementary file 4 — Negative controls for immunocytochemistry. H3K27me3 staining of male (upper) and female (lower) fibroblasts to ensure specific binding of Xi condensation (A). Only secondary antibody staining of iPSCs for Alexa-fluor goat anti mouse 594 and 488 (left) and Alexa-fluor goat anti rabbit 594 and 488 (right)(B). (PPTX 6162 kb) [file 12015_2018_9851_MOESM4_ESM.pptx]

## Slide 1
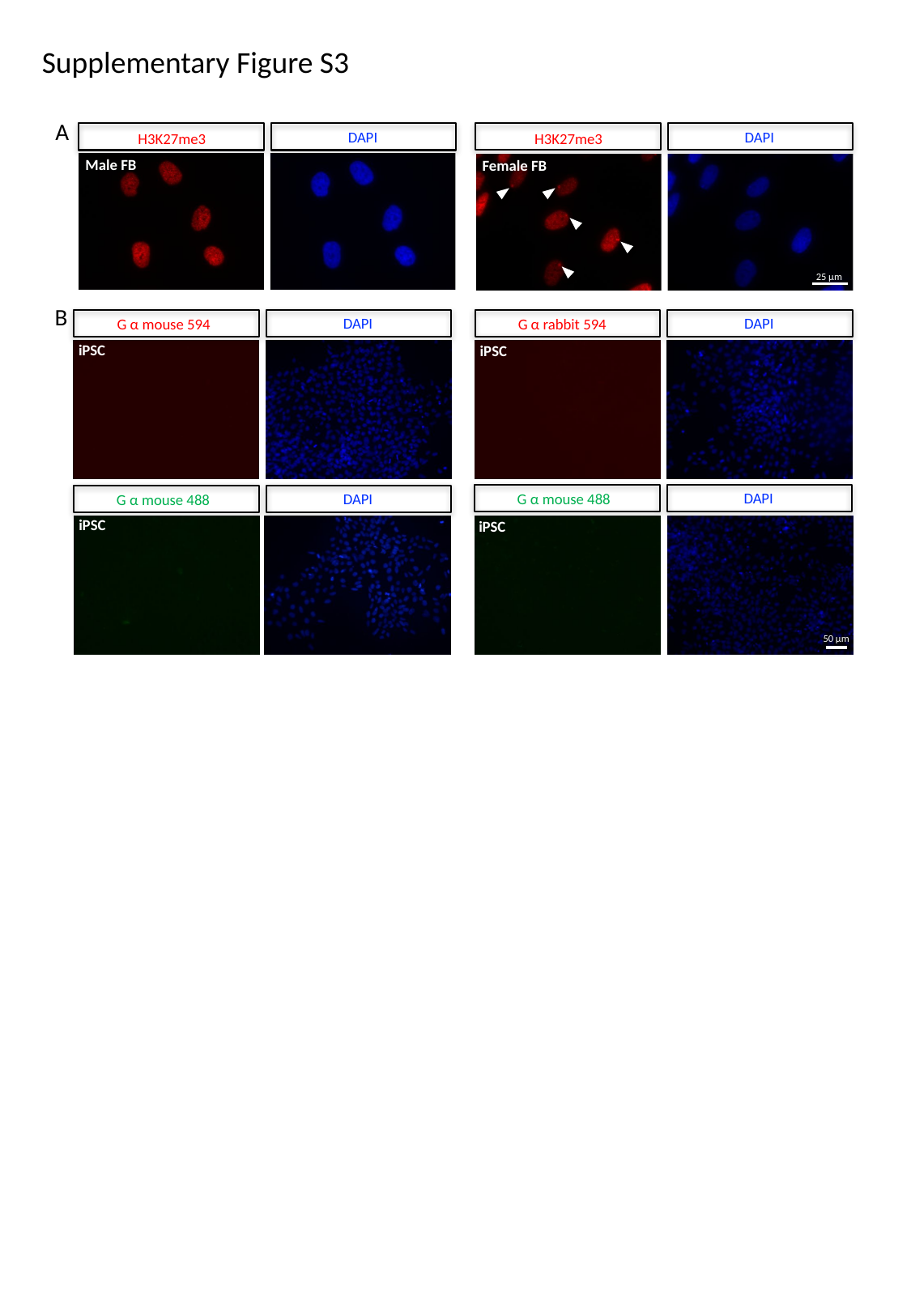

Supplementary Figure S3
A
DAPI
DAPI
H3K27me3
H3K27me3
Male FB
Female FB
25 μm
B
DAPI
DAPI
G α rabbit 594
G α mouse 594
iPSC
iPSC
DAPI
DAPI
G α mouse 488
G α mouse 488
iPSC
iPSC
50 μm
